# A new CECR1 mutation associated with severe hematological involvement in ADA2 deficiency

**DOI:** 10.1002/iid3.930

**Published:** 2023-08-22

**Authors:** Delia Nicoară, Cristina Niță, Ana Stanilă, Alexandru Martiniuc, Laura Popa, Eliana Petrescu, Mihaela Bătăneant, Ruxandra Ciofu, Adriana Guriță, Radu Tabăcaru, Ruxandra Ionescu, Laura Groșeanu

**Affiliations:** ^1^ Sfânta Maria Clinical Hospital Bucharest Romania; ^2^ The Children's Emergency Hospital Loius Turcanu Timișoara Romania; ^3^ Marie Skłodowska Curie Children's Clinical Hospital Bucharest Romania; ^4^ Carol Davila University of Medicine and Pharmacy Bucharest Romania

**Keywords:** adenosine deaminase 2 deficiency, mutation, neutropenia

## Abstract

**Background:**

The deficiency of adenosine deaminase 2 (DADA2) is an autosomal recessively inherited disease resulting from loss‐of‐function mutations in ADA2, formerly named *CECR1* (cat eye syndrome chromosome region, candidate 1) gene. Disease manifestations could be separated into three major phenotypes: inflammatory/vascular, immune dysregulatory, and hematologic; however, most patients presented with significant overlap between these three phenotype groups.

**Case Presentation:**

We present a case of DADA2 deficiency with disease onset at 3 years old, not recognized till the age of 18 with severe gastrointestinal vasculitis and recurrent episodes of neutropenia associated with a new CECR1 mutation.

## Graphical abstract

The diagnosis of DADA2 is often delayed due to its pleiomorphic manifestations: vasculitis, immune dysregulation and variable hematologic involvement.

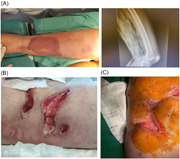

Over the last few years, next‐generation sequencing has led to the detection of more than 50 monogenic autoinflammatory diseases,^1^ among which is the deficiency of adenosine deaminase 2 (DADA2). Associated with biallelic mutations in ADA2 (previously CECR1, cat eye syndrome chromosome region, candidate 1), it was first described in 2014, as a disease similar to polyarteritis nodosa with early‐onset stroke, systemic inflammation, vasculopathy, and mild immunodeficiency.^2^, ^3^ Since then, cumulative reports of approximately 400 patients^4^ have broadened the phenotypic spectrum of this disease ranging from mild skin involvement to major organ involvement^5^ including additional traits such as hematologic defects and features of other rheumatic diseases.^6^, ^7^ Tumor necrosis factor (TNF) inhibition has proven to be highly effective in treating inflammatory manifestations,^8^ while hematopoietic cell transplantation successfully reversed the refractory cytopenia.^9^

We report the fatal case of a female patient with a novel mutation for ADA2 deficiency who displayed isolated severe neutropenia for over 6 years before additional symptoms.

The patient started experiencing recurrent bouts of oral aphthae and fever since the age of 3 years. Around the age of 10, she associated recurrent episodes of oligoarthritis in small and large joints with acute onset. Blood work during the attacks revealed leukopenia (1800–2000 cells/mm^3^) with neutropenia (180/mm^3^) and elevated C‐reactive protein (CRP) (180 mg/L).

At the age of 16, she was diagnosed with Behcet's disease, based on the presence of oral aphthae, one episode of genital aphthae and the presence of HLAB51 antigen. Treatment with short courses of corticosteroids and colchicine was started with a favorable outcome. Complementary investigations showed positive antinuclear antibodies (1:1000) and double‐stranded DNA (up to four times the normal value); thus, the diagnosis was switched to systemic lupus erythematosus (SLE), and she received a low dose of corticosteroids and hydroxychloroquine 200 mg/day daily.

At the age of 17, she was admitted to an emergency department of a pediatric unit for spontaneous clostridial myonecrosis (Figure 1A). Fasciotomy for compartment syndrome, extensive debridement of necrotic tissue, and negative pressure wound dressing therapy saved her life, but during the next months, multiple complications arose (including femoral artery dissection, pelvic abscess, bowel infarction), which led to lower left limb amputation with hip disarticulation, multiple bowel resections, enterocutaneous fistulas, skin grafts, and ileocolostomy with refistulization (Figure 1B). The initial laparotomy revealed a pelvic abscess and two perforations at 15 and 35 cm apart from the ileocecal valve due to bowel infarction. Histopathological examination showed marked inflammatory infiltrates within the lamina with the predominance of lymphoplasmocitar cells (Figure 2). Laparotomy also revealed an ileal stenosis. Repeated episodes (every 2–3 weeks) of severe leukopenia (up to 1300/mm^3^) with neutropenia (up to 20/mm^3^) associated with inflammatory syndrome were noticed during the prolonged hospitalization.

**Figure 1:**
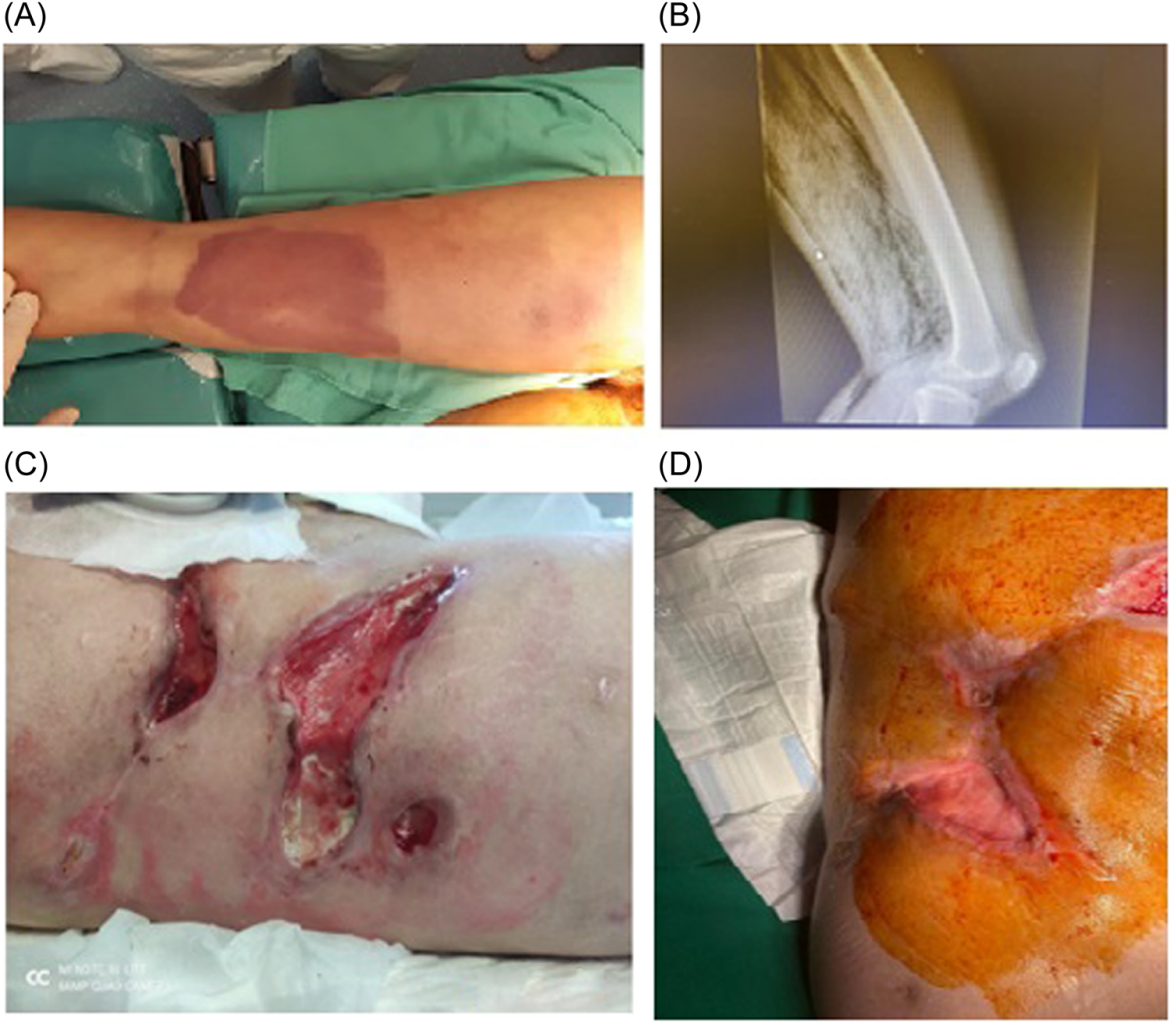
(A) Left lower limb myonecrosis due to *Clostridum septicum*. (B) Radiology proving gas gangrene due to *Clostridium septicum*. (C) Multiple abdominal wounds dehiscences of skin grafts and enterocutaneous fistula. (D) Good evolution of abdominal wall fistula and skin grafts.

**Figure 2:**
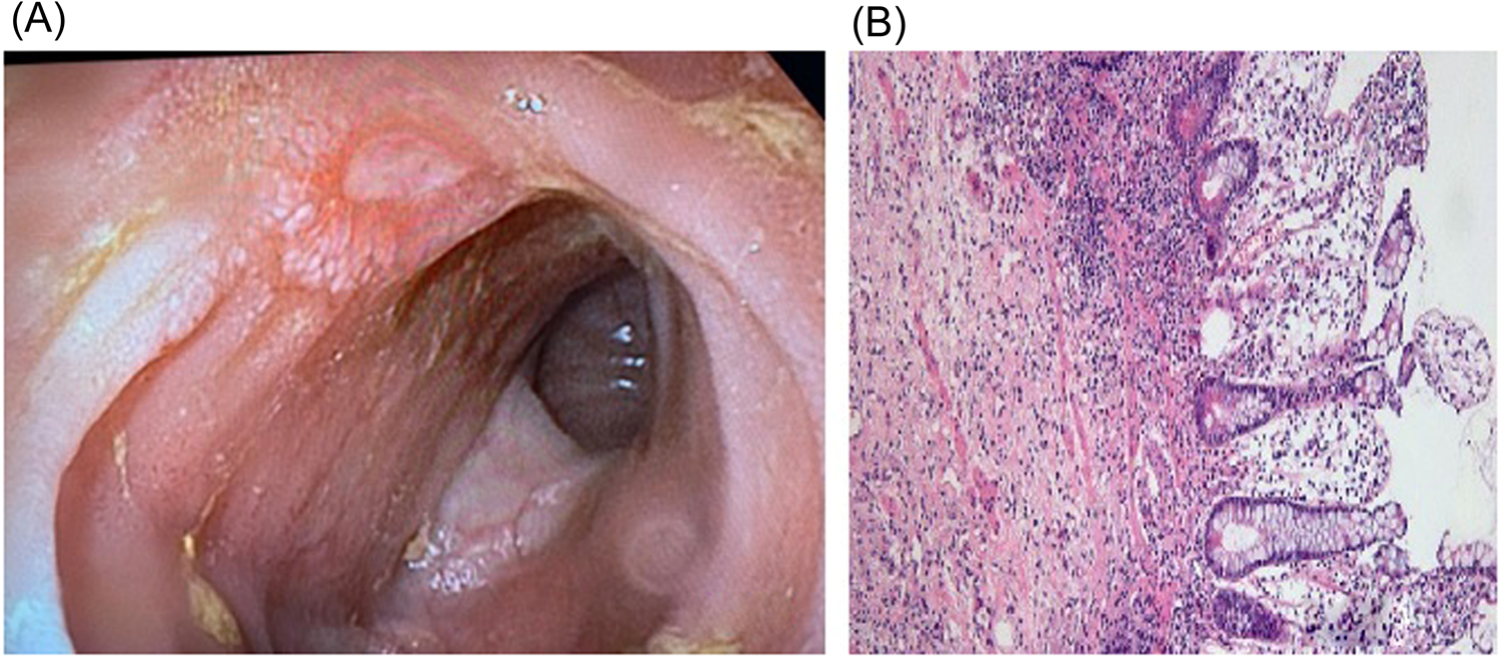
Macroscopic (A) and microscopic (B) appearance of intestinal ulcers.

She was transferred to our clinic with the diagnosis of juvenile SLE with severe hematological and intestinal involvement. Upon arrival in our Rheumatology Unit, lab examination showed mild anemia (10.6 g/dL), mild hypogammaglobulinemia (IgG = 600 mg/dL), normal complement levels, and a slightly elevated ferritin (429 ng/mL). Screening was negative for antinuclear, antiphospholipid, antineutrophil cytoplasmic antibodies, and rheumatoid factor. Peripheral blood flow cytometry showed reversed CD4:CD8 ratios, natural killer cell lymphopenia and decreased peripheral B cells (0.28/mm^3^). Recurrent episodes of neutropenia (up to 700/mm^3^ leukocytes and 2 neutrophils/mm^3^) associated with inflammatory syndrome (erythrocyte sedimentation rate = 80 mm/h, CRP = 130 mg/L) were pointed out, requiring periodic administration of granulocyte‐colony‐stimulating factor 30 MU/0.5 mL daily for 3–5 days and moderate to high doses of corticosteroids with good response (Figure 3).

**Figure 3:**
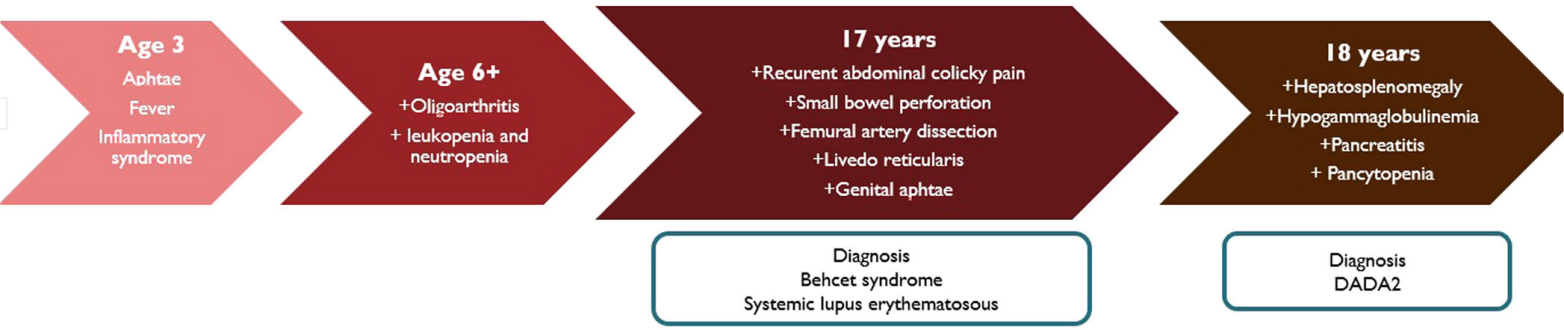
Timeline of events and major clinical manifestations. DADA2, deficiency of adenosine deaminase 2.

Thoracoabdominal computerized tomography (CT) revealed an enterocutaneous fistula and important hepatosplenomegaly; no significant findings were noted on cerebral CT. Angiography could not be performed. Surgical excision of the enteroatmospheric fistula and of the ileocecal pouch was performed with slow to good recovery (Figure 1C).

Many of her clinical features were pointing to Behcet's disease (aphthae, arthritis, intestinal problems), even the presence of HLAB51. However, except for the steroids, no other immunosuppressive worked. Besides that, regular repeated episodes (every 2–3 weeks) of fever, inflammatory syndrome, and leukopenia with neutropenia were pointing to an autoinflammatory disease. Next‐generation sequencing revealed that the patient carried compound heterozygous mutations at positions 139 (exon 2) and 661_664 (exon 4) of the coding sequence of ADA2 (c.139G > A, c.661_664del), resulting, respectively, in amino acid substitution p.Gly47Arg and premature translational stop signal p.Ala221Glnfs*45 in ADA2. Thus, the patient was diagnosed with ADA2 deficiency based on the combination of the clinical picture: cutaneous manifestations (livedo, aphthae), musculoarticular manifestations (arthritis), gastrointestinal manifestations (hepatosplenomegaly, intestinal perforation, possible pancreatitis), hematological manifestation (hypogammaglobulinemia, neutropenia) associated with recurrent fever and inflammatory syndrome and the biallelic mutation of ADA2. ADA2 activity could not be determined. To the best of our knowledge, the second variant was not described in the medical literature so far.

During hospitalization, she experienced multiple infectious episodes—recurrent *Clostridium difficile* colitis, and colonization of surgical wounds with *Klebsiella* and *Pseudomonas*. The clinical picture became even more complicated with an episode of acute interstitial edematous pancreatitis followed by a pancreatic pseudocyst of 6 cm.

When she was stable (normal leukocytes and neutrophils, no inflammatory syndrome, sustained favorable evolution of the surgical wounds), anti‐TNF‐⍺ therapy with etanercept 25 mg was started. Anti‐TNF is the treatment of choice in ADA2 deficiency, being usually safe and effective for most manifestations, although it is often not sufficient for severe hematological phenotypes. At 48 h after the first administration, she developed progressive severe thrombocytopenia (up to 9000/mm^3^), followed shortly by severe pancytopenia. Bone marrow biopsy showed 56% granulocytes, 7%–8% lymphocytes, 1%–2% plasmocytes, 3%–4% blasts, among them 12%–13% promyelocytes, and an increased number of activated macrophages with dense granules. Serum folate and vitamin B12 were normal, and ferritin was slightly elevated (782 ng/mL). Thus, we excluded hemophagocytic lymphohistiocytosis or myelodysplastic syndrome as possible causes. Rising procalcitonin levels (> 10 ng/dL) made sepsis a more plausible cause. Despite supportive treatment, broad‐spectrum antibiotics and antifungal therapy, intravenous immunoglobulins, corticosteroids, erythrocytes, and thrombocyte transfusions, the patient's overwhelming hyperinflammatory syndrome progressed over the next week. This was followed by respiratory deterioration requiring mechanical ventilation and multiorgan failure, which led to the patient's demise the following day. We searched the literature for adverse reactions cited after the initiation of biological treatment; however, severe infections were uncommon following the initiation of anti‐TNF therapy.^4^ Although antiTNF could have triggered the sepsis, we consider that the severe evolution of the disease was the real cause of death. A more aggressive pattern of disease was noticed in the last month of life including new disease manifestations, such as an episode of acute pancreatitis, persistent lymphopenia (50–1300/mm^3^, median 490/mm^3^), and hypogammaglobulinemia nonresponsive to corticosteroids and intravenous immunoglobulins.

Our case highlights the need for early testing in patients with severe inflammatory and hematological manifestations that recur and cannot be controlled with usual therapies and also reports a new mutation possibly associated with severe hematological phenotype of DADA2.

## AUTHOR CONTRIBUTIONS

**Delia Nicoară**: Writing—original draft. **Cristina Niță**: Data curation; methodology; visualization. **Ana Stanilă**: Investigation; validation. **Alexandru Martiniuc**: Investigation; validation. **Laura Popa**: Investigation; validation. **Eliana Petrescu**: Investigation; validation. **Mihaela Bătăneant**: Investigation; methodology; resources. **Ruxandra Ciofu**: Data curation; visualization. **Adriana Guriță**: Funding acquisition; investigation; supervision. **Radu Tabăcaru**: Funding acquisition; investigation; validation. **Ruxandra Ionescu**: Supervision; visualization. **Laura Groșeanu**: Conceptualization; data curation; visualization; writing—review and editing. All authors read and approved the final manuscript.

## CONFLICT OF INTEREST STATEMENT

The authors declare no conflict of interest.

## ETHICS STATEMENT

Local ethics approval was obtained. Written institutional informed consent was obtained from the patient. Written informed consent to publish the case and pictures were obtained from the parents after the patient died.

## Data Availability

The data sets used and/or analyzed during the current study are available from the corresponding author upon reasonable request
